# Are better AI algorithms for breast cancer detection also better at predicting risk? A paired case–control study

**DOI:** 10.1186/s13058-024-01775-z

**Published:** 2024-02-07

**Authors:** Ruggiero Santeramo, Celeste Damiani, Jiefei Wei, Giovanni Montana, Adam R. Brentnall

**Affiliations:** 1 Wolfson Institute of Population Health, Queen Mary University of London, Charterhouse square, London, EC1M 6BQ England, UK; 2 Warwick Manufacturing Group, University of Warwick, Coventry, CV4 7AL England, UK; 3 Fondazione Istituto Italiano di Tecnologia (IIT), 16163 Genoa, Italy; 4 Department of Statistics, University of Warwick, Coventry, CV4 7AL England, UK

**Keywords:** Breast cancer, Risk assessment, AI, Deep learning, Medical imaging

## Abstract

**Background:**

There is increasing evidence that artificial intelligence (AI) breast cancer risk evaluation tools using digital mammograms are highly informative for 1–6 years following a negative screening examination. We hypothesized that algorithms that have previously been shown to work well for cancer detection will also work well for risk assessment and that performance of algorithms for detection and risk assessment is correlated.

**Methods:**

To evaluate our hypothesis, we designed a case-control study using paired mammograms at diagnosis and at the previous screening visit. The study included *n* = 3386 women from the OPTIMAM registry, that includes mammograms from women diagnosed with breast cancer in the English breast screening program 2010–2019. Cases were diagnosed with invasive breast cancer or ductal carcinoma in situ at screening and were selected if they had a mammogram available at the screening examination that led to detection, and a paired mammogram at their previous screening visit 3y prior to detection when no cancer was detected. Controls without cancer were matched 1:1 to cases based on age (year), screening site, and mammography machine type. Risk assessment was conducted using a deep-learning model designed for breast cancer risk assessment (Mirai), and three open-source deep-learning algorithms designed for breast cancer detection. Discrimination was assessed using a matched area under the curve (AUC) statistic.

**Results:**

Overall performance using the paired mammograms followed the same order by algorithm for risk assessment (AUC range 0.59–0.67) and detection (AUC 0.81–0.89), with Mirai performing best for both. There was also a correlation in performance for risk and detection within algorithms by cancer size, with much greater accuracy for large cancers (30 mm+, detection AUC: 0.88–0.92; risk AUC: 0.64–0.74) than smaller cancers (0 to < 10 mm, detection AUC: 0.73–0.86, risk AUC: 0.54–0.64). Mirai was relatively strong for risk assessment of smaller cancers (0 to < 10 mm, risk, Mirai AUC: 0.64 (95% CI 0.57 to 0.70); other algorithms AUC 0.54–0.56).

**Conclusions:**

Improvements in risk assessment could stem from enhancing cancer detection capabilities of smaller cancers. Other state-of-the-art AI detection algorithms with high performance for smaller cancers might achieve relatively high performance for risk assessment.

**Supplementary Information:**

The online version contains supplementary material available at 10.1186/s13058-024-01775-z.

## Introduction

Breast cancer is one of the most prevalent diseases and common cause of death in women worldwide, despite improvements in treatment and mammography screening coverage [1]. Early detection of breast cancer remains an important objective for population health. In recent years, improvements in AI and deep learning technologies have helped to improve the technical accuracy of cancer detection methods [2–4], including for breast cancer detection [5, 6]. Real-world evaluations of how these tools might be used effectively in practice are ongoing [7]. Coupled with these developments for cancer detection are ongoing studies to evaluate risk-based breast cancer screening. This paradigm aims to personalise early detection by directing more intensive screening to those at greatest risk of death from cancer [8]. Retrospective evidence suggests that using AI based on mammograms for risk assessment might be more informative over a 1–6-year period than classical models, and therefore a potentially important component for new risk-based strategies [9–11].

One algorithm that has been evaluated in multiple settings for risk assessment is called Mirai [12]. This deep-learning algorithm was trained for risk assessment using a large US cohort. A limitation of this method compared with classical breast cancer risk models using epidemiological risk factors [13] is that the AI model is largely a black box, and it is not clear why it appears to perform well. In an earlier analysis that we conducted of this algorithm using data from the OPTIMAM repository, we noted that it performed quite well as a cancer detection algorithm, that it was a good predictor of advanced (Stage 2+) cancers, and that the performance and probability of cancer increased as mammograms were taken closer to diagnosis [10]. Other algorithms trained for cancer detection have also shown potential utility for risk assessment, including one called GMIC (globally aware multiple instance classifier) [12, 14]. We therefore hypothesized that algorithms that have previously been shown to work well for cancer detection will also work well for risk assessment and further that performance of algorithms for detection and risk assessment is correlated. In this paper, we report a study to test this hypothesis directly. This is done by evaluating the performance of different algorithms for both detection and risk assessment using paired mammograms on the same women attending screening in the English program, taken at the time cancer was detected at screening, and at a previous screening appointment three years prior to diagnosis when cancer was not detected or detectable by radiologists.

## Methods

### Patients

Patients attended the National Health Service Breast Screening Program (NHSBSP) between February 2010 and September 2019 at sites that are part of the OPTIMAM mammography image database (OMI-DB, see supplementary material and Halling-Brown et al. [15]). The source data are accessible for other research groups. Our manuscript reports new work that has not been undertaken or reported previously using data from this database [16]. Patients were eligible for inclusion if they had standard four-view mammography of ‘for presentation’ type and had normal or malignant episode outcomes. Screening episodes were excluded if the mammograms were not from Hologic machines, or if the woman was not 46–74 year at the time of their screening mammogram, or the woman had breast implants. All mammograms were taken using Hologic Lorad Selenia or Hologic Selenia Dimensions Mammography Systems, following requirements for the MIRAI algorithm [17].

### Algorithms

Four open-source algorithms were applied. They were designed for breast cancer risk assessment, or breast cancer detection. The first algorithm (Mirai version 0.3.1) is a risk assessment algorithm. It is composed of four underlying modules that process each mammogram separately, then combine information before estimating risk annually for 5 years [18]. In our study, we exclusively provided image data as input to the examined algorithm. However, it is also equipped to process additional risk factor data if available. The other selected algorithms were initially designed for the purpose of cancer detection. These were: GMIC [14], and two others from a New York (NY) group that we call: NY [19], and NY-H, where H denotes heatmap and the algorithm is an extension of NY model using heatmaps [19]. Both the NY and NY-H models, along with GMIC, employ deep convolutional neural networks. Among them, GMIC operates with the most intricate architecture. It produces pixel-level saliency maps highlighting potential malignant areas. This innovation finds application in screening mammography interpretation, specifically in predicting the presence of benign or malignant lesions. In contrast, NY-base and NY-H introduce a deep convolutional neural network for breast cancer screening examination classification, employing a two-stage architecture and training approach that strategically combine multiple input views. All three algorithms were trained and evaluated on the same dataset with image-level labels comprising over 225,000 examinations and more than 1 million images. Due to their development process, we a priori expected GMIC to perform better than NY-H for cancer detection and NY-H to perform better than NY. The reason for including all three algorithms is that the expected variation in performance is helpful to test our hypothesis on the correlation between algorithm performance for detection and risk assessment.

### Study design

The target population of our study was women who attended the NHS Breast Screening Program 2010–2019. The primary endpoint was diagnosis of invasive or insitu carcinoma, with biopsy-confirmed cancer diagnoses as recorded on the National Breast Screening System (NBSS). The main predictor variable evaluated was probability of cancer according to the algorithm. For Mirai we used 3 year risk, corresponding to the triennial population screening program. Age range was restricted because most women have screening age 50 to 70 years in England; with some starting aged 47 years or ending aged 73 years during the study epoch due to the age-extension trial (ISRCTN33292440); opportunistic screening is available for those older than 73 years. The case–control study reported is a sub-study of the case–control study reported by [10]. Only screen-detected cancers were included from the earlier work, restricting analysis to matched pairs (case and control) with screening mammograms taken both at diagnosis and 3 years prior to diagnosis (or pseudo diagnosis). The primary focus was on the relationship between performance of predictions by AI algorithms at the time of detection and for risk assessment 3 years prior to detection.

We determined that sample size was sufficient for this analysis before running the algorithms. This was because previous analysis showed a very strong statistical relationship between the Mirai algorithm for both detection and risk [10].

### Statistical analysis

All analysis was adjusted for the site where mammography was done, the model of the mammography device, and where appropriate, age. Predictive performance was measured using the area under the curve (AUC) associated with the algorithm prediction, after adjustment for matching factors with 95% confidence intervals from Wilson’s method [20]. Heterogeneity was assessed using likelihood-ratio tests for interaction based on conditional logistic regression models. The strength of association between risk of breast cancer at diagnosis with risk of breast cancer 3 year prior to diagnosis was evaluated graphically, sub-categorized by tumor size and type (invasive, size unknown; DCIS; invasive: 0 to < 10 mm; 10 to < 20 mm; 20 to < 30 mm; 30 mm+), and by grade (1–2 vs. 3) and estrogen-receptor (ER) status (postive/negative). These cutpoints have been used previously [10]. This was done by estimating model performance using women with mammograms at diagnosis and 3 years prior to diagnosis. A further analysis was conducted by sub-categorizing the data based on the age at which patients were diagnosed with cancer. This stratification involved the utilization of age subgroups (< 55, 55 to 59, 60 to 64, 65 to 69, and 70+), which were chosen to keep the age range in each group relatively constant.

## Results

**Table 1:** Baseline characteristics of participants by case/control status

Summary	Control	Case
Age		
$<55$	400 (24%)	400 (24%)
55–64	896 (53%)	896 (53%)
65+	397 (23%)	397 (23%)
Median (IQR)^a^	60 (55 to 64)	60 (55 to 64)
Site		
Site 1	1247 (74%)	1246 (74%)
Site 2	446 (26%)	447 (26%)
Model		
Lorad Selenia	1643 (97%)	1643 (97%)
Selenia dimensions	50 (3%)	50 (3%)
Cancer		
Insitu		420 (25%)
Invasive		1263 (75%)
Unknown		10 (1%)

^a^*IQR* inter-quartile range

Women were included in this analysis from the previous case–control study if they had mammograms at detection (or pseudo detection if controls) and their previous screening visit 3 years earlier. This led to complete data on *n* = 3386 cases and controls, matched 1:1. Basic demographic characteristics of those included in the study, including matching variables, are shown in Table 1.

**Fig. 1:**
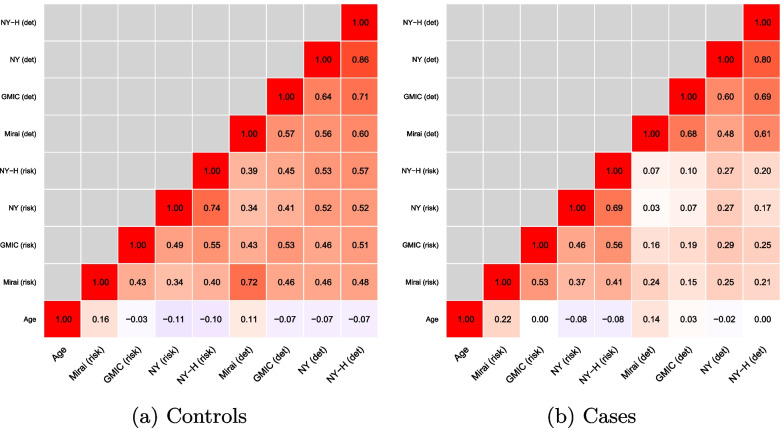
Spearman correlation between algorithms when used on mammograms 3 year prior to diagnosis (risk) or at diagnosis (det)

**Fig. 2:**
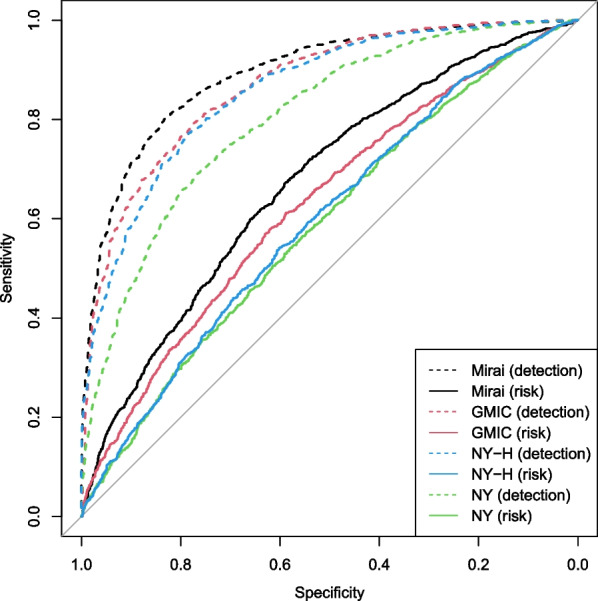
Receiver operating characteristics for detection and risk by algorithm

Figure 1 reports Spearman correlation between the algorithms in controls and cases. In controls, there was only a weak correlation between the algorithm prediction and age. Correlation was moderate to good between algorithms. Mirai was more correlated with itself for detection and risk than the other three algorithms; the NY and NY-H for risk were more correlated with each other than with Mirai or GMIC, as might be expected due to their similar architecture and process of development. It is also of interest that there was a higher correlation between Mirai and age, than the other algorithms. Because age is a strong risk factor for breast cancer, and Mirai was trained for risk assessment, it appears that it uses age from mammography scans for risk assessment, whereas the other algorithms developed for cancer detection do not. A different correlation structure was observed in the cases. Here the algorithms were more correlated between themselves for risk or detection, than with themselves for the other set of mammograms. Overall, there was again moderate to good correlation between the algorithms, but also some variation indicating lack of agreement in the rank ordering of patients.

**Fig. 3:**
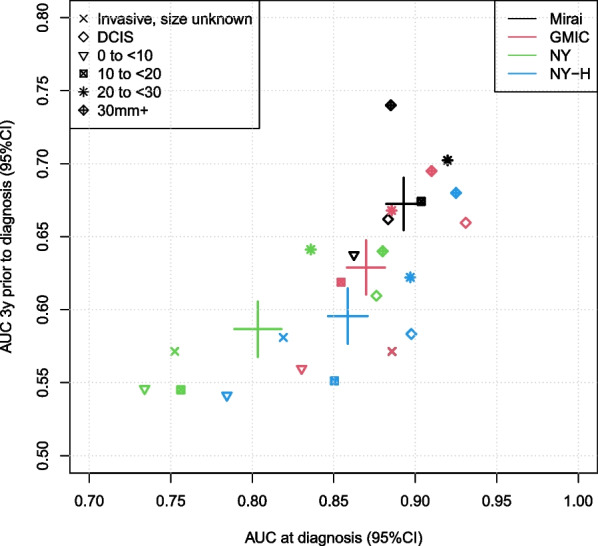
Association between AUC for diagnosis and AUC for risk by algorithm (Mirai black, GMIC red, NY green, NY-H blue) overall ($+$, with width of the bars corresponding to the 95% CI) and by type and size of cancer when detected

The association between algorithm performance for risk and detection is examined further in Table 2 and Fig. 3. Model performance for detection was consistently better for larger cancers across all algorithms. In addition, both within cancer types and size, and across algorithms, there was a pattern whereby better performance for cancer detection was associated with better performance for risk assessment. A similar but less strong pattern was seen by age subgroup (Additional file 1: Fig. S1). On average, the algorithm performed best for risk in the age groups where it performed best for detection. Similar findings were observed by ER subgroups (Additional file 1: Fig. S2) and for cancer grade 1 and 2 (Additional file 1: Fig. S3). However, there was very little association seen for grade 3 cancers, suggesting that different radiological features are observed at detection than at the previous screening mammogram in faster-growing grade 3 cancers.

Figure 2 plots receiver operating characteristics (ROCs) for risk and detection by algorithm. The same ordering of performance by algorithm for both is seen, with Mirai having strongest performance for both risk assessment and detection, and the NY algorithm the weakest.

**Table 2:** Performance of algorithms for detection and risk overall, by cancer type and tumor size

Subgroup	*n*	Detection AUC (95% CI)	Risk AUC (95% CI)	P_het_(detection)	P_het_(risk)
Mirai					
Overall	3386	0.89 (0.88 to 0.91)	0.67 (0.65 to 0.69)		
Size					
Invasive, size unknown	210 (6%)	0.89 (0.81 to 0.93)	0.57 (0.48 to 0.66)	< 0.001	< 0.001
DCIS	840 (25%)	0.88 (0.85 to 0.91)	0.66 (0.62 to 0.71)		
0 to < 10 mm	436 (13%)	0.86 (0.81 to 0.90)	0.64 (0.57 to 0.70)		
10 to < 20 mm	976 (29%)	0.90 (0.87 to 0.93)	0.67 (0.63 to 0.71)		
20 to < 30 mm	524 (15%)	0.92 (0.88 to 0.95)	0.70 (0.64 to 0.75)		
30 mm+	400 (12%)	0.88 (0.83 to 0.92)	0.74 (0.68 to 0.80)		
GMIC					
Overall	3386	0.88 (0.87 to 0.90)	0.63 (0.61 to 0.66)		
Size					
Invasive, size unknown	210 (6%)	0.89 (0.81 to 0.93)	0.57 (0.48 to 0.66)	< 0.001	< 0.001
DCIS	840 (25%)	0.93 (0.90 to 0.95)	0.66 (0.61 to 0.70)		
0 to < 10 mm	436 (13%)	0.83 (0.77 to 0.87)	0.56 (0.49 to 0.62)		
10 to < 20 mm	976 (29%)	0.85 (0.82 to 0.88)	0.62 (0.57 to 0.66)		
20 to < 30 mm	524 (15%)	0.89 (0.84 to 0.92)	0.67 (0.61 to 0.72)		
30 mm+	400 (12%)	0.91 (0.86 to 0.94)	0.70 (0.63 to 0.75)		
NY					
Overall	3386	0.81 (0.79 to 0.83)	0.59 (0.57 to 0.61)		
Size					
Invasive, size unknown	210 (6%)	0.75 (0.66 to 0.83)	0.57 (0.48 to 0.66)	< 0.001	< 0.001
DCIS	840 (25%)	0.88 (0.84 to 0.90)	0.61 (0.56 to 0.65)		
0 to < 10 mm	436 (13%)	0.73 (0.67 to 0.79)	0.55 (0.48 to 0.61)		
10 to < 20 mm	976 (29%)	0.76 (0.72 to 0.79)	0.55 (0.50 to 0.59)		
20 to < 30 mm	524 (15%)	0.84 (0.79 to 0.88)	0.64 (0.58 to 0.70)		
30 mm+	400 (12%)	0.88 (0.83 to 0.92)	0.64 (0.57 to 0.70)		
NY-H					
Overall	3386	0.87 (0.85 to 0.88)	0.59 (0.56 to 0.61)		
Size					
Invasive, size unknown	210 (6%)	0.82 (0.73 to 0.88)	0.58 (0.49 to 0.67)	< 0.001	< 0.001
DCIS	840 (25%)	0.90 (0.86 to 0.92)	0.58 (0.54 to 0.63)		
0 to < 10 mm	436 (13%)	0.78 (0.73 to 0.83)	0.54 (0.47 to 0.61)		
10 to < 20 mm	976 (29%)	0.85 (0.82 to 0.88)	0.55 (0.51 to 0.59)		
20 to < 30 mm	524 (15%)	0.90 (0.85 to 0.93)	0.62 (0.56 to 0.68)		
30 mm+	400 (12%)	0.92 (0.88 to 0.95)	0.68 (0.61 to 0.74)

## Discussion

Our analysis suggests that algorithms that perform better for cancer detection are also likely to perform better for risk assessment. We found evidence that “easier to detect” larger cancers at diagnosis are also likely to be given a higher probability of malignancy three years prior to diagnosis, i.e., for risk assessment.

There are several implications of our findings. Firstly, improvements for risk assessment algorithms for breast cancer might be gained by improving their performance for cancer detection. For example, analysis by subgroup of cancer size showed that GMIC was broadly comparable with Mirai at detection of larger cancers (> 10 mm) and DCIS, but worse for smaller cancers both at diagnosis and at risk assessment. To achieve similar performance for risk assessment therefore, one might suggest additional training or developments of the GMIC algorithm to focus on smaller cancers—improvements to risk assessment are likely to follow. Secondly, our results suggest that state-of-the-art algorithms for breast cancer detection might be considered to be repurposed for risk assessment. In time, AI for mammography is likely to become implemented in national screening programs such as the UK. Such developments could then enable routine risk assessment to help drive new risk-based screening regimens. Thirdly, our findings help to explain why Mirai works well for risk assessment: it is finding early signs of cancer. These are likely most visible in the larger cancers at screen detection because they are more likely to have been there at the previous screen than smaller cancers which might have only developed in the interval between screens. Fourthly, our results suggest that, more generally, deep learning computer vision algorithms are able to discern intricate patterns in breast scans, which are not currently acted upon by radiologists. Their ability to extract latent insights from visual data only without the use of any classical risk factors suggests that the development of a more sophisticated diagnostic models should yield better results for risk assessment.

Strengths of our study include the paired design, whereby the mammograms at detection and earlier screening rounds were on the same women. Our design has not been used before to assess correlation between performance for detection and risk assessment. It is also little applied in other work on AI algorithms for breast cancer risk assessment, where most publications have focused on cancer following a single screening visit for risk assessment. Using paired data lets us test our hypothesis more reliably than indirect comparisons of performance for risk and detection using samples of different women. Another strength is that this study was an external validation assessment of all the algorithms, with no training or fine tuning done. This helps to ensure a reliable evaluation.

There are several limitations to our study. Firstly, although some algorithms produce heatmaps that can provide a more in-depth view of the inner mechanisms, the algorithms were applied as a “black box”, and we do not know if the higher risk was due to a suspicious area in the region where the cancer was found, or something else. For example, an alternative explanation for the findings might be that the algorithms identify a field effect in the breast, not a specific pattern associated with breast cancer. Secondly, our study is a retrospective and observational case–control study. The area is largely lacking evaluation through more prospective designs, and the retrospective nature of this work makes it at risk of bias including related to the decision to seek publication of results. Thirdly, the analysis is limited by when and where screening mammograms were recorded (e.g., it was based on women attending the English screening program, but we do not know the race or ethnicity of those included). Fourthly, we were unable to compare directly with other domains or risk models, including family history and polygenic risk scores; or the other risk factors that may be added to Mirai. Fifthly, we were limited by availability of code to run pre-defined algorithms for risk or detection. Other algorithms may perform differently, and this is worth further investigating. Lastly, it is important to note that this study specifically focused on mammograms acquired via Hologic machines, which may constrain the applicability of the findings to other types of mammogram machines.

In conclusion, this study evaluated whether the performance of an AI model for detection is associated with its performance for risk assessment. We did this using four open-source algorithms. The analysis suggests that algorithms that excel at cancer detection also perform well for risk assessment. The correlation between the ability to detect cancer in mammograms and the ability to assess the risk of developing cancer suggests that improvements in risk assessment algorithms could be obtained by focusing on improving their capabilities for cancer detection. For instance, algorithms may need additional training on detecting smaller cancers to achieve better performance in risk assessment. More generally, current state-of-the-art detection algorithms might be repurposed for risk assessment. This could enable the AI technologies currently being trialled to aid cancer detection using mammograms to play a vital role in future risk-based screening programs. For example, it might be advisable to recommend more frequent screening for higher-risk patients [21]. Finally, the paired mammograms in our study were about 3 years apart as per the standard breast cancer screening interval in the UK. Therefore, the evidence reported is most relevant to short-term breast cancer risk, perhaps due to the detection of indolent breast cancers not detected by the human eye. To better inform long-term mammography screening patterns, developing breast cancer risk prediction models over a longer time horizon would be useful. This extension could provide a comprehensive understanding of breast cancer risk dynamics and contribute to refining strategies for effective and personalized long-term screening.

### Supplementary Information

**Additional file 1**. Supplementary Figures, Methods and Tables.

## Data Availability

The images and data used in this publication are derived from the OPTIMAM imaging database [15], we would like to acknowledge the OPTIMAM project team and staff at the Royal Surrey NHS Foundation Trust who developed the OPTIMAM database, and Cancer Research UK who funded the creation and maintenance of the database.
