# Supplementary material for: Are better AI algorithms for breast cancer detection also better at predicting risk? A paired case–control study
Source: Breast Cancer Res. 2024 Feb 7;26:25. doi: 10.1186/s13058-024-01775-z (PMC10848404; doi:10.1186/s13058-024-01775-z)
Supplement: Supplementary file 1 — Additional file 1. Supplementary Figures, Methods and Tables. [file 13058_2024_1775_MOESM1_ESM.pdf]

A Supplementary material

A.1 Methods

The OMI-DB is a mammography image database comprised of unprocessed and processed full-field digital mammograms. It collects NHSBSP images from multiple breast screening centres across the UK, with the goal of serving as a large repository of medical images for research and training purposes. As part of the data sharing agreement, we obtained a subset of this database composed by data of 18,800 patients, of which (before data cleaning):

- 5500 malignant cases with annotations
- 600 benign cases with annotations
- 800 malignant cases
- 1000 benign cases
- 900 interval cancer cases
- 10000 normal cases

For most imaging events (for example, a screening is an imaging event) we have processed and unprocessed full-field digital mammograms, and two views of each breast, i.e. medio-lateral oblique (MLO) and cranio-caudal (CC). The database contains images from different manufacturers, particularly Hologic Inc (models Hologic Lorad Selenia and Selenia Dimensions Mammography Systems), and General Electric (GE) Medical Systems (models Senograph DS and Senographe Essential), Siemens and Philips. The images were collected from three sites, and not all manufacturers were only used at each site.

Table S1: Performance of algorithms for detection and risk by cancer type and age.

Subgroup	n	Detection AUC (95%CI)	Risk AUC (95%CI)	Phet (detection)	Phet (risk)
Mirai					
Overall	3386	0.89 (0.88 to 0.91)	0.67 (0.65 to 0.69)		
Cancer type					
Insitu	840 (25%)	0.88 (0.85 to 0.91)	0.66 (0.62 to 0.71)	0.33	0.6
Invasive	2526 (75%)	0.90 (0.88 to 0.91)	0.68 (0.65 to 0.70)		
Unknown	20 (1%)	0.70 (0.40 to 0.89)	0.50 (0.24 to 0.76)		
Age					
<55	802 (24%)	0.86 (0.82 to 0.89)	0.62 (0.57 to 0.67)	0.48	0.035
55-64	1794 (53%)	0.90 (0.88 to 0.92)	0.69 (0.66 to 0.72)		
65+	790 (23%)	0.90 (0.87 to 0.93)	0.67 (0.62 to 0.72)		
GMIC					
Overall	3386	0.88 (0.87 to 0.90)	0.63 (0.61 to 0.66)		
Cancer type					
Insitu	840 (25%)	0.93 (0.90 to 0.95)	0.66 (0.61 to 0.70)	0.014	0.9
Invasive	2526 (75%)	0.87 (0.85 to 0.88)	0.63 (0.60 to 0.65)		
Unknown	20 (1%)	1.00 (0.72 to 1.00)	0.60 (0.31 to 0.83)		
Age					
<55	802 (24%)	0.87 (0.83 to 0.90)	0.63 (0.58 to 0.67)	0.8	0.8
55-64	1794 (53%)	0.90 (0.88 to 0.92)	0.65 (0.62 to 0.68)		
65+	790 (23%)	0.87 (0.84 to 0.90)	0.61 (0.56 to 0.66)		
NY					
Overall	3386	0.81 (0.79 to 0.83)	0.59 (0.57 to 0.61)		
Cancer type					
Insitu	840 (25%)	0.88 (0.84 to 0.90)	0.61 (0.56 to 0.65)	0.23	0.9
Invasive	2526 (75%)	0.79 (0.76 to 0.81)	0.58 (0.55 to 0.61)		
Unknown	20 (1%)	0.80 (0.49 to 0.94)	0.80 (0.49 to 0.94)		
Age					
<55	802 (24%)	0.81 (0.77 to 0.85)	0.59 (0.54 to 0.64)	0.28	0.6
55-64	1794 (53%)	0.82 (0.80 to 0.85)	0.60 (0.56 to 0.63)		
65+	790 (23%)	0.78 (0.73 to 0.81)	0.57 (0.52 to 0.62)		
NY-H					
Overall	3386	0.87 (0.85 to 0.88)	0.59 (0.56 to 0.61)		
Cancer type					
Insitu	840 (25%)	0.90 (0.86 to 0.92)	0.58 (0.54 to 0.63)	1.0	0.9
Invasive	2526 (75%)	0.86 (0.84 to 0.88)	0.59 (0.56 to 0.61)		
Unknown	20 (1%)	0.80 (0.49 to 0.94)	0.60 (0.31 to 0.83)		
Age					
<55	802 (24%)	0.85 (0.81 to 0.88)	0.59 (0.54 to 0.64)	0.15	0.25

55-64	1794 (53%)	0.88 (0.85 to 0.90)	0.60 (0.57 to 0.63)
65+	790 (23%)	0.87 (0.83 to 0.90)	0.55 (0.50 to 0.60)

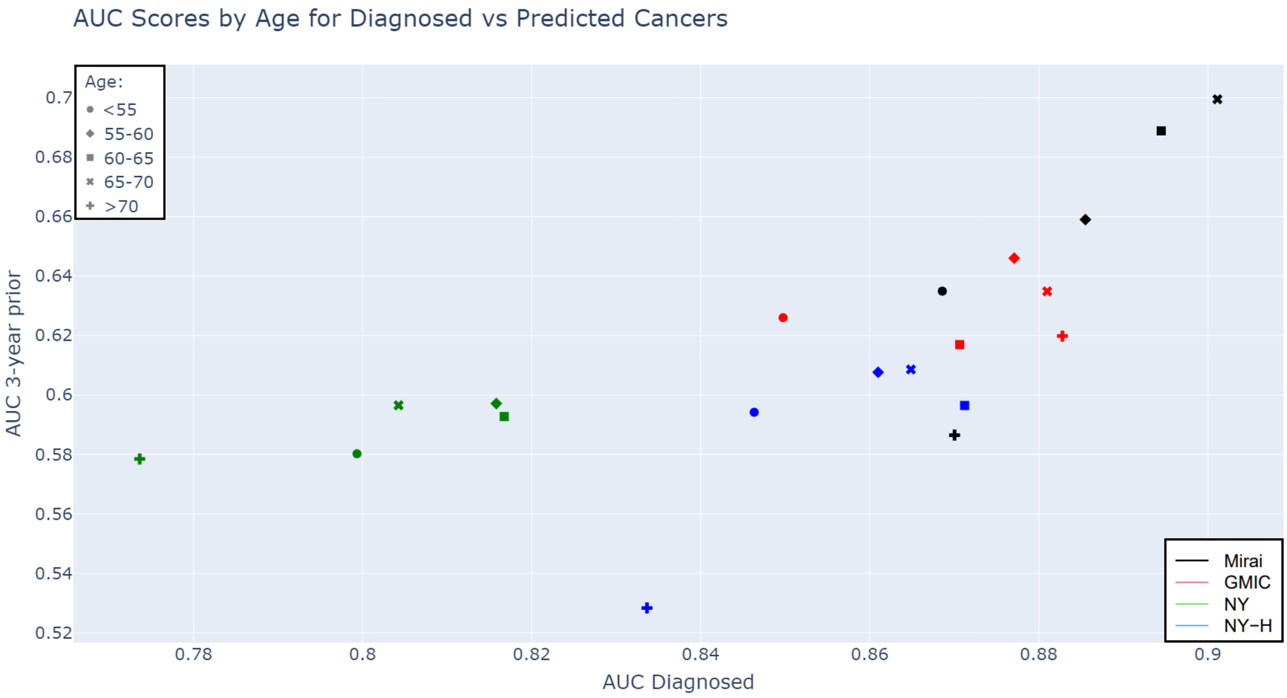

Figure S1: Association between AUC computed at diagnosis and AUC computed at 3-year prior for risk by algorithm (Mirai black, GMIC red, NY green, and NY-H blue) and age of patient when cancer was detected.

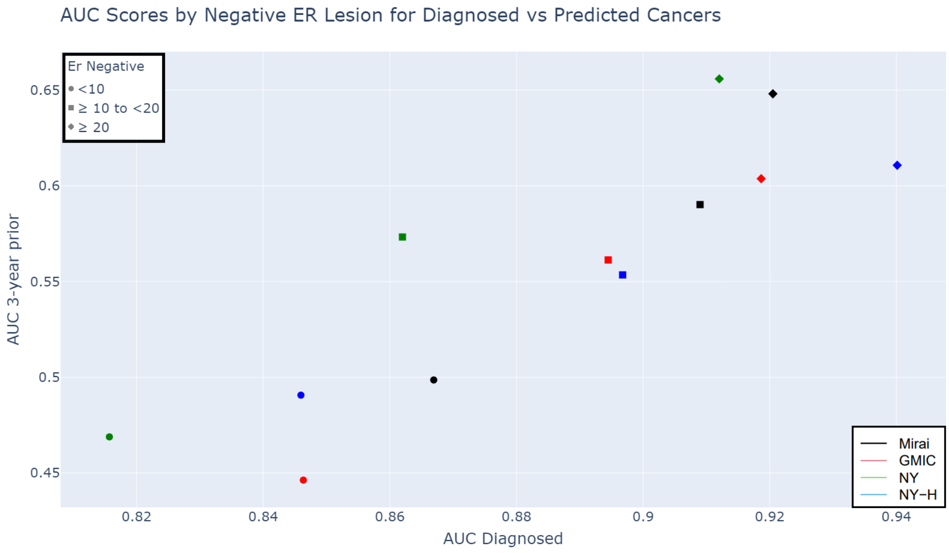

(a) Negative ER Lesion Status

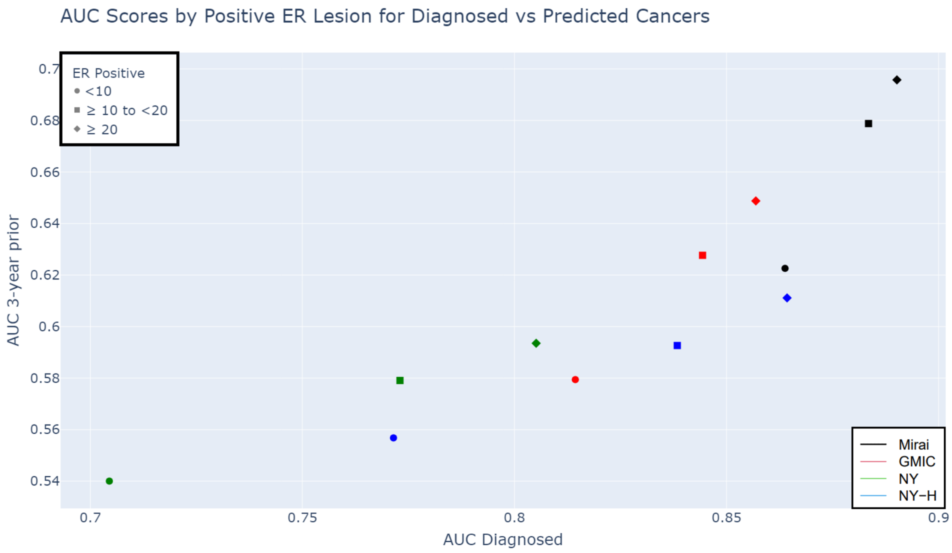

(b) Positive ER Lesion Status

Figure S2: Association between AUC computed at diagnosis and AUC computed at 3-year prior for risk by algorithm (Mirai black, GMIC red, NY green, and NY-H blue) and size of patient when cancer was detected with negative (up) and positive (down) ER status.

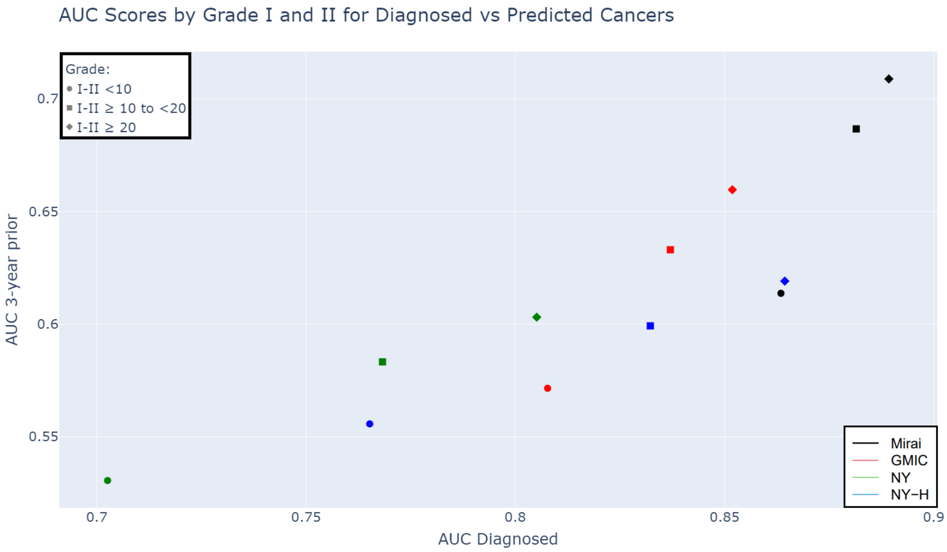

(a) Cancer with Grade I and II

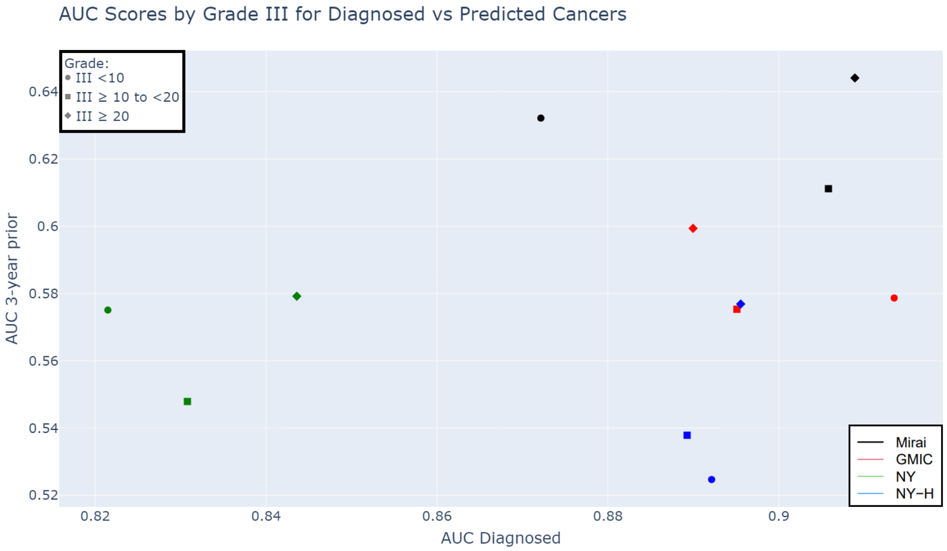

(b) Cancer with Grade III

Figure S3: Association between AUC computed at diagnosis and AUC computed at 3-year prior for risk by algorithm (Mirai black, GMIC red, NY green, and NY-H blue) and size of patient when cancer was detected with Grade I and II (up) and Grade III (down).
